# Changing effects of energy and water on the richness distribution pattern of the *Quercus* genus in China

**DOI:** 10.3389/fpls.2024.1301395

**Published:** 2024-01-17

**Authors:** Shuxia Sun, Yang Zhang, Naixian Wang, Wenjun Yang, Yinuo Zhai, Hui Wang, Peixian Fan, Chao You, Peiming Zheng, Renqing Wang

**Affiliations:** ^1^ Institute of Ecology and Biodiversity, School of Life Sciences, Shandong University, Qingdao, China; ^2^ Shandong Provincial Engineering and Technology Research Center for Vegetation Ecology, Shandong University, Qingdao, China; ^3^ Qingdao Forest Ecology Research Station of National Forestry and Grassland Administration, Shandong University, Qingdao, China; ^4^ Qingdao Key Laboratory of Forest and Wetland Ecology, Shandong University, Qingdao, China; ^5^ Department of Statistics and Actuarial Science, Northern Illinois University, Dekalb, IL, United States

**Keywords:** climatic determinants, energy and water, geographically weighted regression, *Quercus* genus, richness distribution pattern

## Abstract

Climate varies along geographic gradients, causing spatial variations in the effects of energy and water on species richness and the explanatory power of different climatic factors. Species of the *Quercus* genus are important tree species in China with high ecological and socioeconomic value. To detect whether the effects of energy and water on species richness change along climatic gradients, this study built geographically weighted regression models based on species richness and climatic data. Variation partition analysis and hierarchical partitioning analysis were used to further explore the main climatic factors shaping the richness distribution pattern of *Quercus* in China. The results showed that *Quercus* species were mainly distributed in mountainous areas of southwestern China. Both energy and water were associated with species richness, with global slopes of 0.17 and 0.14, respectively. The effects of energy and water on species richness gradually increased as energy and water in the environment decreased. The interaction between energy and water altered the effect of energy, and in arid regions, the effects of energy and water were relatively stronger. Moreover, energy explained more variation in species richness in both the entire study area (11.5%) and different climate regions (up to 19.4%). The min temperature of coldest month was the main climatic variable forming the richness distribution pattern of *Quercus* in China. In conclusion, cold and drought are the critical climatic factors limiting the species richness of *Quercus*, and climate warming will have a greater impact in arid regions. These findings are important for understanding the biogeographic characteristics of *Quercus* and conserving biodiversity in China.

## 1 Introduction

The distribution pattern of biodiversity and its underlying mechanisms and determinants are one of the central issues in macroecology and biogeography (Gaston, 2000; Lomolino et al., 2017; McGill, 2019). The contemporary climate, environmental heterogeneity, historical events, and biological evolution work together to shape biodiversity (O'Brien et al., 2000; Wiens and Donoghue, 2004; Kreft and Jetz, 2007; Allouche et al., 2012; Stein et al., 2014; Willig and Presley, 2018; Dimitrov et al., 2023). Among these factors, contemporary climate is one of the major factors affecting the distribution pattern of biodiversity at broad scales (Wang et al., 2011; Chen et al., 2015; Vázquez-Rivera and Currie, 2015). Species richness, which refers to the number of species in a given habitat, is regarded as an important dimension of biodiversity (Nagalingum et al., 2015; Molles and Sher, 2018; Willig and Presley, 2018; Wang et al., 2022). Numerous studies have also confirmed that climate plays a decisive role in shaping species richness, especially energy (i.e., temperature) and water (i.e., precipitation) (O'Brien, 2006; Eiserhardt et al., 2011; Wang et al., 2011; Vázquez-Rivera and Currie, 2015; Xu et al., 2019; Subedi et al., 2020).

It has been widely demonstrated that species richness is strongly correlated with energy and water (Hawkins et al., 2003; Field et al., 2009; Brown, 2014; Lomolino et al., 2017; Bohdalková et al., 2021; Coelho et al., 2023). Warmer and more humid regions provide more available resources to plants, eventually sustaining higher species richness (Jiménez-Alfaro et al., 2016; Pandey et al., 2020). Bhatta et al. (2021) found that the richness distribution pattern of plants across the Himalayas could be explained by the interaction of energy, water, and their seasonal variation. Xu et al. (2019) reported that the species richness of the *Quercus* genus was a function of energy, water, and their interaction. China is a vast country with diverse climate zones (ranging from tropical to boreal zones) and vegetation types (including deserts, grasslands, alpine meadows, and forests), leading to obvious gradients of energy and water (Fang et al., 2002; Ding, 2013). There are consequent geographical shifts in species richness and spatial variations in the effects of energy and water (Hawkins et al., 2014). However, only a small number of studies have focused on these changing effects along climatic gradients on the regional scale (Coelho et al., 2023).

The gradients of energy and water also cause the explanatory power of different climatic factors for species richness to change across regions (Svenning et al., 2009). Previous research has explored the richness distribution patterns of different taxa and their climatic determinants in China (Chen et al., 2011; Wang et al., 2011; Shrestha et al., 2018; Chen and Su, 2020; Wang et al., 2022). For example, Pandey et al. (2020) clarified that climatic seasonality was the predominant factor underlying the distribution pattern of gymnosperm richness in China and found that energy–water was the best set to predict changes in species richness. Shrestha et al. (2018) studied the drivers of high *Rhododendron* diversity in southwest China and confirmed that climatic gradients and temperature seasonality played a key role in determining *Rhododendron* diversity. However, there is still a lack of research to identify the main climatic factors forming the richness distribution pattern of the *Quercus* genus in China and quantify their explanatory powers.

Species of the *Quercus* genus are important tree species all over the world, especially in the northern hemisphere (Menitsky, 2005; Gil-Pelegrín et al., 2017; Subedi et al., 2020; Chen et al., 2021; Xia et al., 2022). *Quercus* species are also dominant species that constitute 10% of China’s forest and have high ecological and socioeconomic value, such as maintaining biodiversity, ensuring ecosystem stability, and providing fire protection (Zhang, 2007; Gil-Pelegrín et al., 2017; National Forestry and Grassland Administration, 2019; Xia et al., 2022). According to the Flora of China Editorial Committee (1999), there are 40 species in the *Quercus* genus in China. These species are widely distributed, with high species diversity and broad ecological amplitudes, making *Quercus* a suitable taxon for large-scale research (Flora of China Editorial Committee, 1999; Zhang, 2007; Xu et al., 2016).

Based on species richness and climatic data, the present study examined how spatial variations in energy and water influenced the richness distribution pattern of the *Quercus* genus in China with geographically weighted regression (GWR). The relative importance of different climatic factors for determining the species richness of the *Quercus* genus was also calculated using variation partition analysis. This study aimed to answer the following questions: (1) What is the richness distribution pattern of the *Quercus* genus in China? (2) Do the effects of energy and water on species richness change along climatic gradients? (3) Which climatic factor is the most important in shaping the richness distribution pattern? Taking *Quercus* species distributed in China as research objects and investigating the relationship between species richness and climate are of great significance for understanding the biogeographic characteristics of the *Quercus* genus and conserving biodiversity in China.

## 2 Materials and methods

### 2.1 Species richness data

Thirty-five species in the *Quercus* genus distributed in China were selected for this research. The checklist was derived from the *Flora of China*, excluding three varieties and two species of the *Cyclobalanopsis* genus (**Supplementary Table S1**) (Flora of China Editorial Committee, 1999). Species distribution data were obtained from two sources: online specimen databases (the Chinese Virtual Herbarium database, http://www.cvh.ac.cn/; the National Specimen Information Infrastructure database, http://www.nsii.org.cn/2017/home.php; the PE Herbarium database, http://pe.ibcas.ac.cn/) and field surveys. Because most specimen records were recorded by administrative divisions or geographic locations and lacked information on the latitude and longitude, all species distribution data were organized at the county level, and only one of the multiple data located in the same unit (one county) was retained for each species. The number of species in a unit was used as the species richness data. To avoid the impact of the area on the research, the species richness data were converted to 20’ × 20’ grid cells (Zhang et al., 2017; Zhang et al., 2019). A total of 3,676 grid cells containing species richness data were utilized for analysis.

### 2.2 Climatic data

A frequently used climatic dataset from the WorldClim database (version 2.1, https://www.worldclim.org/data/worldclim21.html) was chosen for this study (Xu et al., 2019; Sun et al., 2020; Dimitrov et al., 2023; Poppenwimer et al., 2023; Yang et al., 2023). This dataset contained 19 bioclimatic variables, of which BIO1–BIO11 represented energy availability, while BIO12–BIO19 represented water availability (**Table 1**). In addition, the potential evapotranspiration (PET), actual evapotranspiration (AET), and aridity index (AI) were selected as research variables and downloaded from the CGIAR consortium for spatial information (http://www.cgiar-csi.org/). PET can characterize energy in an area, AET reflects the amount of water actually available to plants, and AI is an indicator assessing drought conditions (Wang et al., 2009; Li et al., 2013; Zomer et al., 2022). All climatic variables are listed in **Table 1**. The climatic data were resampled with a spatial resolution matching the accuracy of rasterized species richness data to 20 × 20 arc minutes in ArcGIS 10.6.

**Table 1:** Climatic variables used in this study.

Variable	Abbreviation	Unit
Energy		
Annual mean temperature	BIO1	°C
Mean diurnal range	BIO2	°C
Isothermality (BIO2/BIO7)×100	BIO3	/
Temperature seasonality (SD×100)	BIO4	°C
Max temperature of warmest month	BIO5	°C
Min temperature of coldest month	BIO6	°C
Temperature annual range	BIO7	°C
Mean temperature of wettest quarter	BIO8	°C
Mean temperature of driest quarter	BIO9	°C
Mean temperature of warmest quarter	BIO10	°C
Mean temperature of coldest quarter	BIO11	°C
Potential Evapotranspiration	PET	mm
Water		
Annual precipitation	BIO12	mm
Precipitation of wettest month	BIO13	mm
Precipitation of driest month	BIO14	mm
Precipitation seasonality	BIO15	/
Precipitation of wettest quarter	BIO16	mm
Precipitation of driest quarter	BIO17	mm
Precipitation of warmest quarter	BIO18	mm
Precipitation of coldest quarter	BIO19	mm
Actual Evapotranspiration	AET	mm
Aridity Index	AI	/

To analyze the problem of multi-collinearity between climatic variables, Spearman correlation analysis was performed for the variables of energy and water. The results showed significant correlations between climatic variables (**Supplementary Figure S1**). Principal component analysis (PCA) was performed for energy and water variables separately to address the problem of multi-collinearity. According to the stopping rules in PCA, the first principal components (PC1) for energy (EPC1) and water (WPC1) were extracted to represent the gradients of energy and water after dimensionality reduction, respectively (Jackson, 1993).

### 2.3 Data analysis

Ordinary least square (OLS) regression was employed to examine the relationship between the species richness of the *Quercus* genus and the climate in China. The OLS regression assumed a stationary relationship within the research area and generated global slopes on behalf of the constant effects of energy and water on species richness (Jetz et al., 2005; Weisberg, 2005; Hawkins et al., 2011; Li et al., 2013; Suissa et al., 2021). Then, GWR was conducted to determine whether the richness–climate relationship shifted geographically. GWR took the spatial non-stationarity into account, introduced distance weights, and regressed at each specific geographic location, enabling the estimation of local slopes in each study unit (one grid cell) and the assessment of the changing effects of energy and water on species richness (Fotheringham et al., 2002; Charlton et al., 2009; Eiserhardt et al., 2011; Keith et al., 2014; Huang et al., 2023). The local slope signified how many species could be gained or lost when changing one unit of energy or water (Xu et al., 2016). Two GWR models were constructed, with one (the GWR energy model) including species richness and EPC1, and the other (the GWR water model) including species richness and WPC1, and acquired local slopes indicating the effects of energy (Eslopes) and water (Wslopes) on species richness.

The model performance was estimated based on the correlated Akaike information criterion (AIC_C_) value, where the smaller the value, the better the model (Cavanaugh, 1997; Fotheringham et al., 2002). It is generally accepted that there is a significant difference between models when the AIC_C_ values differ by more than three (Cavanaugh, 1997). Through the residual distribution of the OLS model, whether there was a spatial variation in the relationship between species richness and climate could be determined (Tripathi et al., 2019). This was the prerequisite for subsequent research.

Two linear models were established to evaluate the changing effects of energy and water along climatic gradients, with Eslopes against EPC1 and Wslopes against WPC1. Furthermore, regression analyses were conducted with Eslopes/Wslopes/Richness as dependent variables against EPC1 and WPC1 as independent variables to explore how the interaction between energy and water changed the independent effects of these factors on species richness. The results were presented in the form of contour plot.

To identify the main climatic factors shaping the richness distribution pattern of the *Quercus* genus in China, variation partition analysis (VPA) was used to calculate the explanatory powers of energy and water for species richness (Quinn and Keough, 2002; Murray and Conner, 2009; Pandey et al., 2020). As aforementioned, significant correlations between variables were observed (**Supplementary Figure S1**). With the least absolute shrinkage and selection operator (LASSO) algorithm, a common compression estimation method, two sets of variables (representing energy and water) that had a major influence on the species richness with low collinearity were screened in advance and utilized in VPA (Efron et al., 2004; Sun et al., 2020). Next, the relative importance of the selected variables was quantified using hierarchical partitioning (HP) analysis (Mac-Nally, 2002; Mac-Nally and Walsh, 2004; Pandey et al., 2020).

Based on the results of PCA for energy and water variables, the scores on the first axis were divided into four gradients (according to the quartiles): 0–25%, 25%–50%, 50%–75%, and 75%–100%, corresponding to the cold/arid, cool/semi-arid, warm/semi-humid, and hot/humid regions, respectively. The procedures above were repeated in different climate regions.

OLS and GWR were modeled in ArcGIS 10.6, and the value of species richness was log-transformed. The Lasso algorithm, VPA, and HP analysis were performed with R software. Other analyses were conducted in Origin 2022.

## 3 Results

### 3.1 Climate and richness distribution patterns

**Figure 1:**
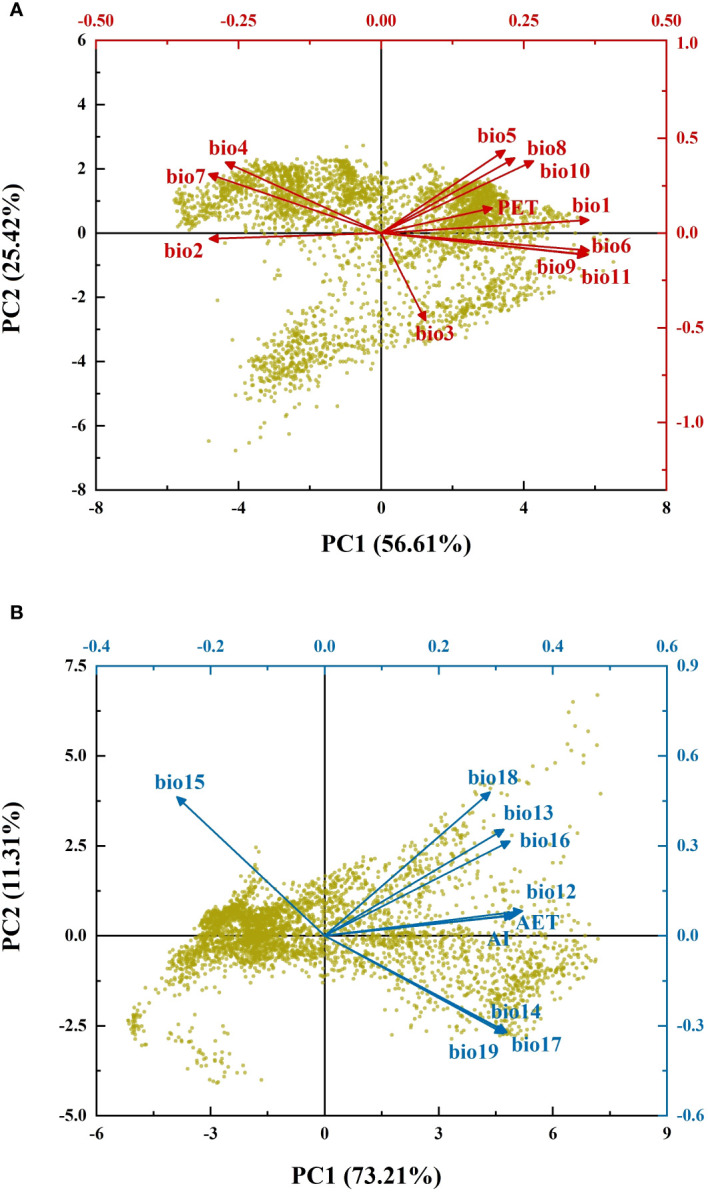
Biplot of principal component analysis. **(A)** Principal component analysis of energy variables; **(B)** principal component analysis of water variables. Black axes represent the loading plots, while red and blue axes represent the score plots.

**Figure 2:**
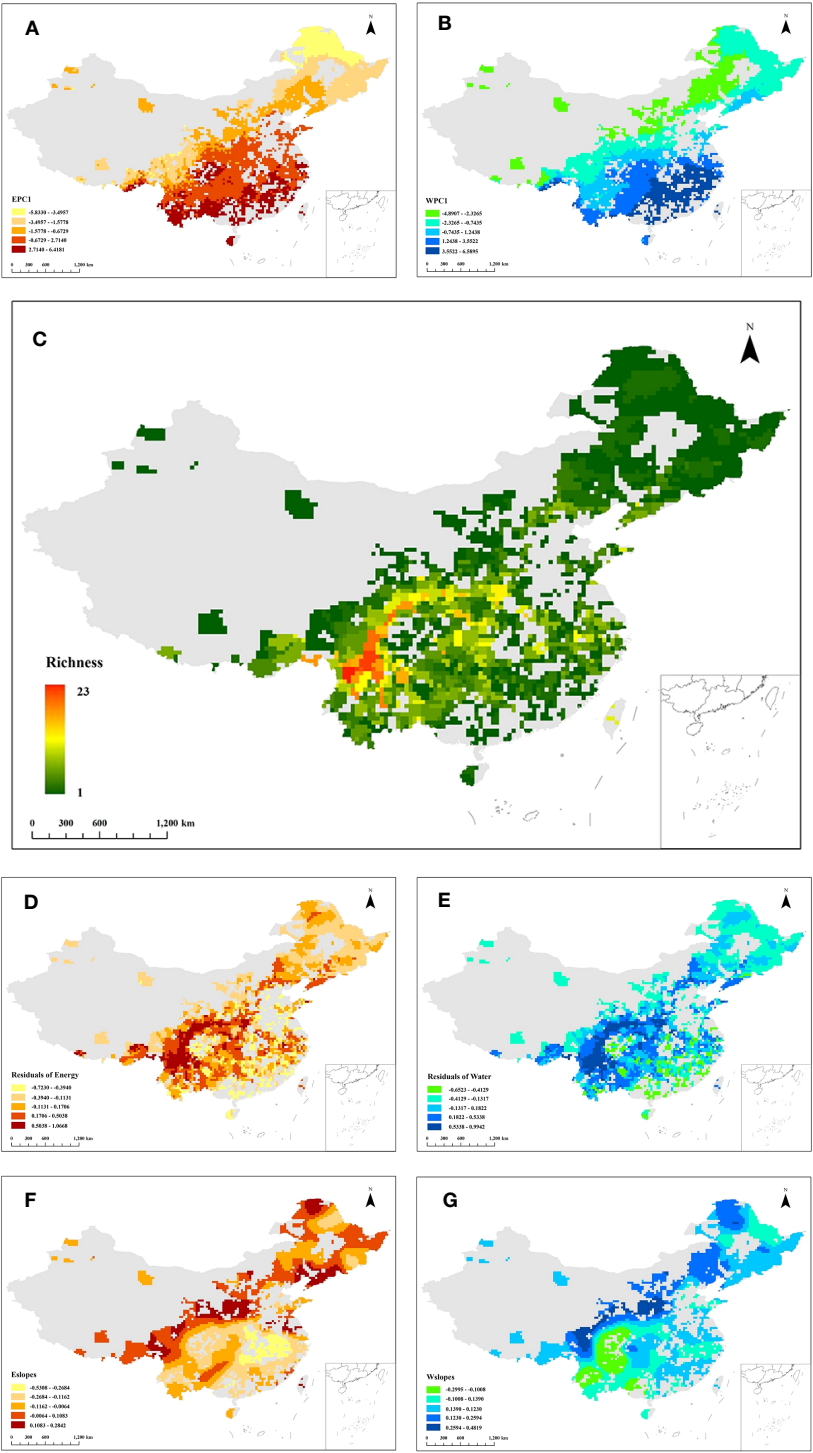
Distribution pattern of first principal component for **(A)** energy variables (EPC1) and **(B)** water variables (WPC1). **(C)** Richness distribution pattern of the *Quercus* genus in China. Colors from red to green represent species richness from high to low. Residual distribution pattern of the OLS energy model **(D)** and OLS water model **(E)**. **(F)** Local slopes of the GWR energy model (Eslopes) distribution pattern. **(G)** Local slopes of the GWR water model (Wslopes) distribution pattern. The grey region in all figures represents the area without the distribution of *Quercus* species.

The results of PCA showed that the first principal components for energy (EPC1) and water variables (WPC1) explained 56.61% and 73.21% of the variance, respectively (**Figure 1**). According to the broken-stick stopping principle, EPC1 and WPC1 contained most of the information of the original climatic variables and could reflect variations in energy and water. **Figures 2A, B** depict the distribution patterns of EPC1 and WPC1, respectively. The distribution of climate exhibited an evident spatial pattern. With increasing latitude, the ambient energy (EPC1) gradually decreased. In addition to the change in latitude, there was also a reduced trend of available water (WPC1) from coastal to inland areas. This was consistent with the current climatic conditions in China.

Species of the *Quercus* genus are widely distributed in China, but the richness distribution is uneven. The species richness of *Quercus* is higher in the south of China than in the northern region. As presented in **Figure 2C**, *Quercus* species are mainly concentrated in the mountains of southwestern China, and the area with the highest species richness is located in the Hengduan Mountains, where the species richness reaches 23, accounting for 65.71% of the total number of *Quercus* species.

### 3.2 Establishment, evaluation, and selection of models

The results of OLS model revealed that both energy and water were associated with the species richness of the *Quercus* genus in China, with global slopes of 0.17 (p < 0.01) and 0.14 (p < 0.01), respectively. Comparing the adjusted R^2^ and AIC_C_ values showed that energy and water variables were better fitted by the GWR model than the OLS model (**Table 2**). The residual distribution of the OLS model also exhibited a clear spatial pattern, demonstrating that the relationship between species richness and climate existed geographical shifts (**Figures 2D, E**). Therefore, the results of the GWR model were chosen for the subsequent analysis.

**Table 2:** Different parameters derived from the OLS and GWR models.

Model	R^2^	Adjusted R^2^	AIC_C_	ΔAIC_C_
**OLS energy model**	0.14	0.14	2830.05	2594.02
**GWR energy model**	0.58	0.57	236.03	
**OLS water model**	0.10	0.10	3004.63	2515.79
**GWR water model**	0.55	0.54	488.84	

### 3.3 Changing effects of energy and water on species richness

**Figure 3:**
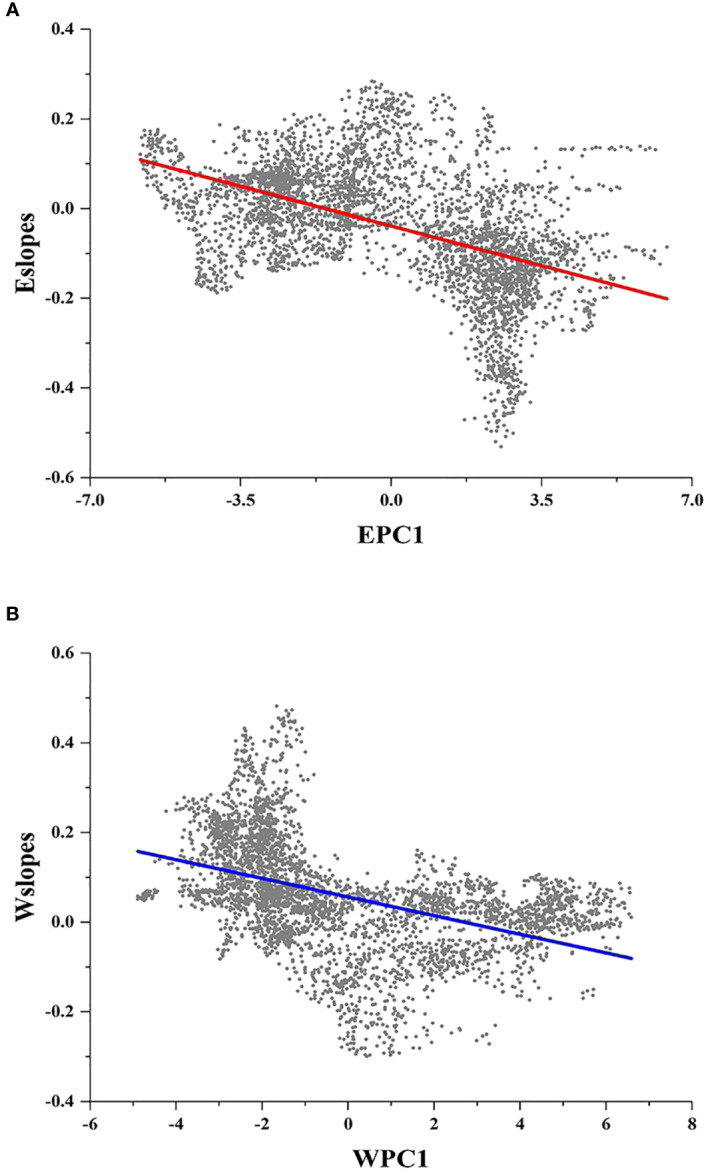
Local slopes of the GWR model change along climate gradients. **(A)** Local slopes of the GWR energy model (Eslopes) decrease significantly along energy gradients (EPC1) (R^2^ = 0.245; P < 0.001). **(B)** Local slopes of the GWR water model (Wslopes) decrease significantly along water gradients (WPC1) (R^2^ = 0.206; P < 0.001).

**Figure 4:**
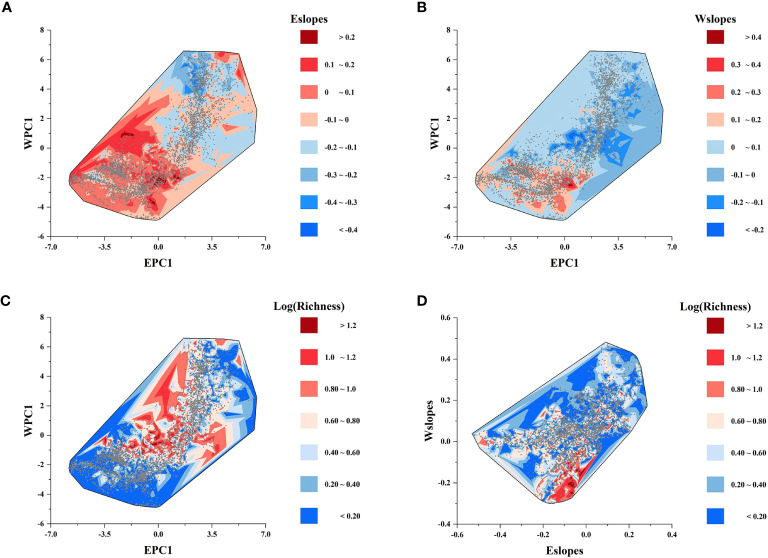
Contour plots showing the effect of the interaction between energy and water on **(A)** the local slopes of the GWR energy model (Eslopes); **(B)** the local slopes of the GWR water model (Wslopes); and **(C)** species richness. **(D)** Variation of species richness along the gradients of the local slopes of the GWR energy model (Eslopes) and GWR water model (Wslopes).

**Figures 2F, G** display the local slopes of the GWR energy model (Eslopes) and the GWR water model (Wslopes) distribution patterns, indicating apparent spatial variations in the effects of energy and water on the species richness of *Quercus* in China. Both Eslopes and Wslopes exhibited significant declines along with the increase in energy and water in the environment (**Figure 3**). Contour plots indicated that the interaction between energy and water had a certain effect on Eslopes. The decreasing trend of Eslopes with increasing EPC1 became indistinct, and the effect of energy on species richness was highest when energy availability was at a medium level, which signified that Eslopes would be influenced by water availability as it changed along EPC1 gradients (**Figure 4A**). The energy–water interaction had little effect on Wslopes. The gradual decline in Wslopes along WPC1 gradients is still distinct in **Figure 4B**, confirming that the shift of Wslopes with WPC1 is not affected by energy availability. As shown in **Figures 4C, D**, the highest species richness occurred in the areas with moderate energy and sufficient water, and the effect of climate on the species richness was the lowest at this time.

**Figure 5:**
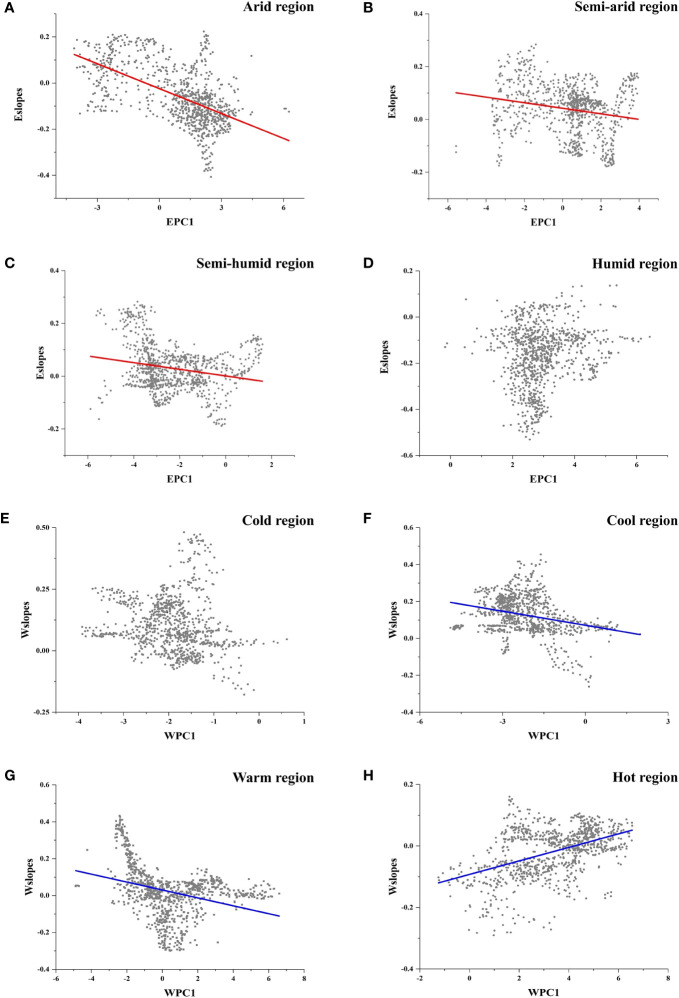
**(A–D)** Local slopes of the GWR energy model (Eslopes) changing along energy gradients in different climate regions. **(A)** Arid regions; **(B)** semi-arid regions; **(C)** semi-humid regions; **(D)** humid regions. **(E–H)** Local slopes of the GWR water model (Wslopes) changing along water gradients in different climate regions. **(E)** Cold regions; **(F)** cool regions; **(G)** warm regions; **(H)** hot regions.

For different climate regions, Eslopes decreased faster with the increase in EPC1 in arid regions (**Figure 5**, **Table 3**), and the values of Eslopes were relatively higher in arid regions (**Figure 4A**). There was no obvious pattern in the variation of Wslopes with water gradients in different climate regions (**Figure 5**, **Table 3**). However, it was worth noting that Wslopes significantly increased with rising WPC1 in hot regions, and the values of Wslopes shifted from negative to positive (**Figure 5H**, **Table 3**).

**Table 3:** Relationship between the local slopes of the GWR model and energy/water gradients in different climate regions.

Relationship	Climate Region	Slope	R^2^	P-value
**Eslopes and EPC1**	Arid	−0.036	0.33	<0.001
**Eslopes and EPC1**	Semi-arid	−0.011	0.14	<0.001
**Eslopes and EPC1**	Semi-humid	−0.013	0.16	<0.001
**Eslopes and EPC1**	Humid	–	–	>0.05
**Wslopes and WPC1**	Cold	–	–	>0.05
**Wslopes and WPC1**	Cool	−0.025	0.15	<0.001
**Wslopes and WPC1**	Warm	−0.021	0.29	<0.001
**Wslopes and WPC1**	Hot	0.022	0.21	<0.001

### 3.4 Main climatic factors and their explanatory powers

**Figure 6:**
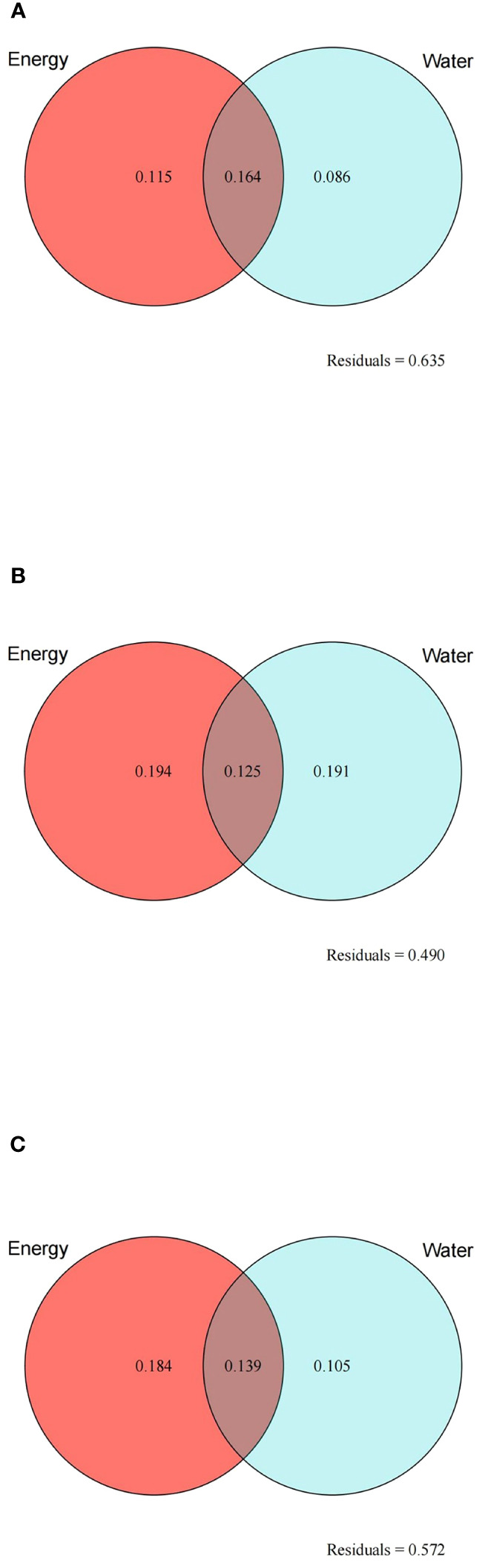
Results of variation partition analysis for the species richness of the *Quercus* genus in China with two sets of variables: energy (red circles) and water (blue circles). **(A)** All study areas; **(B)** cold regions; **(C)** arid regions. Numbers represent the proportion of variation explained (%) by energy and water. The number in the intersection of the two circles represents the joint effect of energy and water. Residuals represent the unexplained variation.

**Figure 7:**
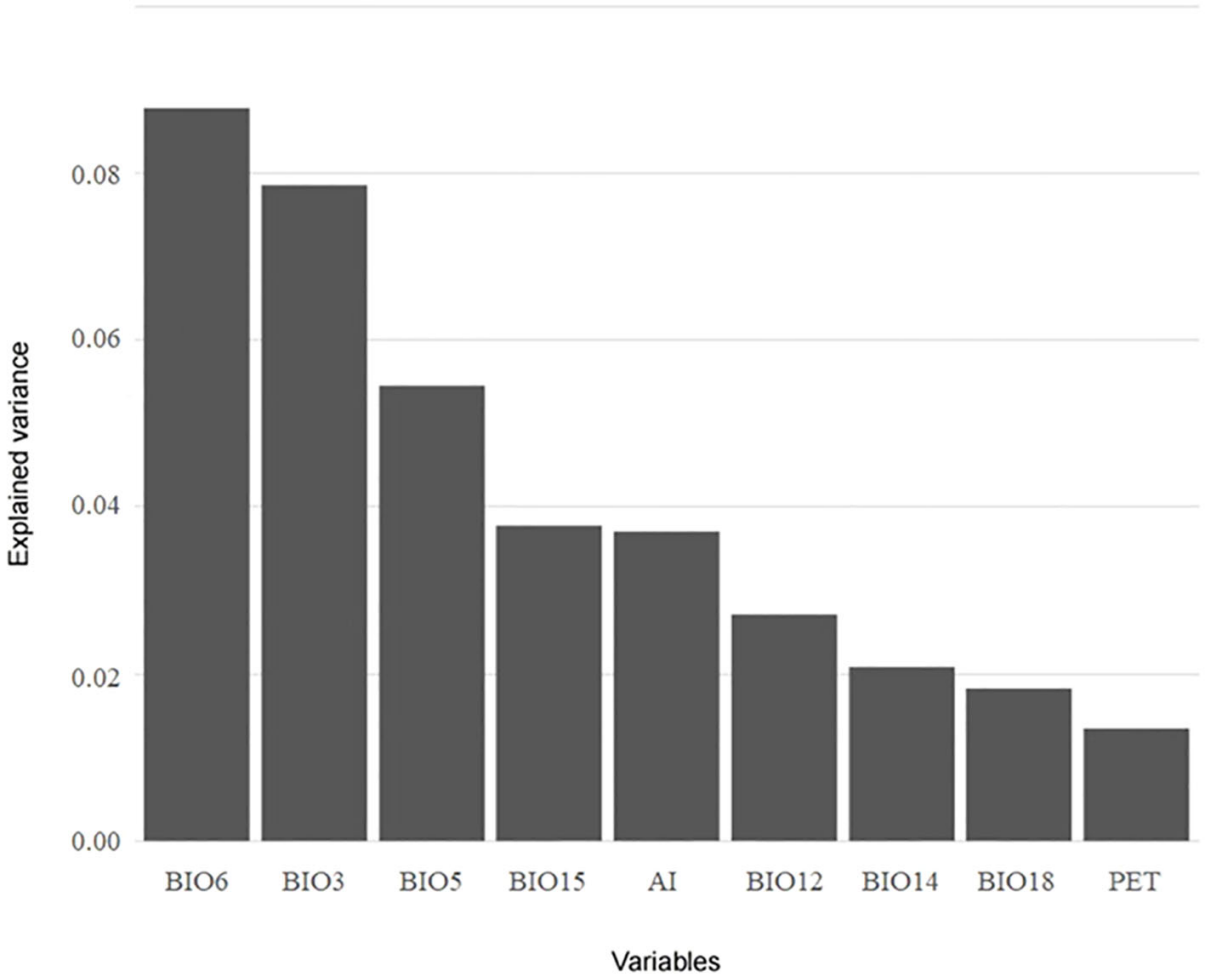
Results of hierarchical partitioning analysis for the species richness of the *Quercus* genus in China with variables screened using the LASSO algorithm.

A total of nine variables, namely BIO3, BIO5, BIO6, and PET on behalf of energy and BIO12, BIO14, BIO15, BIO18, and AI on behalf of water, were screened for VPA and HP analysis using the LASSO algorithm. Based on the results of VPA, the joint effect of energy and water had the highest explanatory power for species richness (16.4%), followed by the independent effect of energy (11.5%) (**Figure 6A**). For cold regions, the explanatory power of different climatic factors increased, with the explanatory power of energy (19.4%) being slightly higher than that of water (19.1%) (**Figure 6B**). In arid regions, the variations explained by energy and water increased (18.4% and 10.5%, respectively), but the independent effect of water remained lower than the joint effect of energy and water (13.9%) (**Figure 6C**). HP analysis provided the relative importance of each variable (**Figure 7**). BIO6 (min temperature of coldest month) made the highest contribution to species richness among all variables, while BIO15 (precipitation seasonality) contributed the most to species richness among water variables. In general, the relative importance of energy variables was higher than that of water variables.

## 4 Discussion

### 4.1 Richness distribution pattern of the *Quercus* genus in China

The richness distribution pattern of the *Quercus* genus in China exhibited great spatial variation, and the areas with high species richness were primarily distributed in southwestern China, mostly along the major mountain ranges (from Hengduan Mountains to Qinling Mountains and Daba Mountains in central China). These mountainous areas with suitable temperatures and stable rainfall are hotspots for the distribution of many species, including the *Quercus* genus (Liu et al., 2017; Zheng et al., 2018; Yang et al., 2023). For example, Xie et al. (2022); Pandey et al. (2020); Sun et al. (2020), and Yu et al. (2017) have reported that the southwestern mountains in China are hotspots for the distribution of threatened conifers, gymnosperms, oaks, and rhododendrons, respectively. The results also indicated that the species distribution of the *Quercus* genus exhibited a certain trend of clustering, which was consistent with the result of a previous study by Sun et al. (2020).

As a whole, the species richness of *Quercus* in northern China was lower than that in southern China. This might be due to the fact that most *Quercus* species originated from tropical or temperate forests and have adapted to warm environments and lack tolerance for coldness (Flora of China Editorial Committee, 1999; Menitsky, 2005; Xu et al., 2016). The northwestern region of China (the Qinghai–Tibet Plateau and most of Inner Mongolia) has harsh and extreme climate conditions (Zhai et al., 1999; Ding, 2013). These areas belong to the temperate desert zone, temperate steppe zone, or Qinghai–Tibet Plateau high cold vegetation zone in terms of vegetation regionalization in China and are not suitable for the growth of *Quercus* species (Fang et al., 2002; Ding, 2013; Li et al., 2013). Therefore, there is basically no record of the species distribution of the *Quercus* genus in these regions (Zhang, 2007).

### 4.2 Changing effects of energy and water on species richness

A positive relationship was found between the species richness of *Quercus* and the climate in China, verifying that increases in energy and water would promote regional species richness. The results also confirmed that the effects of energy and water on species richness varied spatially, with a gradual increase from hot and humid regions to cold and arid regions. This is in accordance with previous findings that cold and drought are the key climatic constraints of species diversity (Xu et al., 2016; Zhang et al., 2019; Huang et al., 2023). It can be inferred that this is due to the enhanced physiological filtering of the environment on species as cold and drought intensify. Low temperatures and water deficits inhibit photosynthesis, slow water transportation, reduce the metabolic rate, and limit the growth of plants (Cavender-Bares et al., 2005; Apostol et al., 2007; Nobel, 2009; Anderegg and HilleRisLambers, 2016; Ahmad et al., 2019; Liu et al., 2021; Liu et al., 2023). By acting on the physiological tolerance of species, the decline in energy and water restricts their geographical distributions and ultimately influences the richness distribution pattern of plants through population dynamics (Anderegg and HilleRisLambers, 2016; Xu et al., 2016; Sun et al., 2020). Vessella and Schirone (2013) determined that drought and cold stresses were the main factors affecting cork oak occurrence in Italy. Xu et al. (2013) examined the role of evolutionary history on the species diversity of oaks and found that niche conservatism extended the effects of low temperature and drought on plant physiology to species distribution and diversity. This is in line with the physiological tolerance hypothesis, which proposes that lower species richness in cold and arid regions is attributed to fewer species being able to adapt to extreme climate conditions (Currie et al., 2004; Wang et al., 2011). This hypothesis was also supported by one of the findings of the present study that the species richness of *Quercus* was generally higher in the south of China than in the northern region.

The results also revealed that the effect of energy on species richness was changed by energy–water interaction. The findings suggest that the closer the available water to the physiological tolerance limit of *Quercus* species, the faster the species richness responds to the ambient energy. Xu et al. (2016) investigated the effect of the interaction between energy and water on the species diversity of *Quercus* and discovered that the effect of energy shifted from positive in dry regions to negative in wet regions, which also demonstrated that water influenced the relationship between diversity and energy. By contrast, the present research did not detect a change in the effect of water on species richness due to energy–water interactions. The gradually increasing effect of water along the gradient of water in hot regions, which was contrary to the overall trend, as well as the negative to positive conversion in the value of the water effect might be on account of the alleviation of water deficiency induced by high temperature.

By dividing the research area into different climate regions, this work demonstrated that the effects of energy and water on species richness were relatively stronger in arid regions. Many studies have corroborated that drought is a determining factor restricting the distributions of certain plants and regional species diversity (Choat et al., 2012; Zhang et al., 2019). Using pteridophytes to test the water–energy dynamic hypothesis, Huang et al. (2023) deduced that shifts of energy and water in dry environments could give rise to dramatic changes in the species richness of pteridophytes. Zhang et al. (2014) predicted that the loss of woody plants in Yunnan Province for the years 2070–2099 would mainly be related to the reduction of precipitation in the dry season and the instability of temperature. Because of global warming, temperature is increasing and will continue to rise in the future (IPCC, 2023). In view of the results of this work, it is speculated that the effect of climatic warming on the species richness of the *Quercus* genus will exhibit spatial differences in China and these species will be more vulnerable to climate change in arid regions. Therefore, it is necessary to strengthen conservation measures in arid regions to reduce the disadvantage of future climate change and lower the risk of species extinction.

### 4.3 Main climatic factors in shaping the richness distribution pattern

Energy explained more variation than water for the species richness of *Quercus* in China in both the entire study area and in different climate regions. Woody plants are more susceptible to energy because they have evolved an improved ability to adapt to water deficits (Oliver et al., 2000; Chen et al., 2015). In particular, most *Quercus* species are drought-resistant and have a relatively high tolerance for water deficits (Flora of China Editorial Committee, 1999; Menitsky, 2005). Compared with other climate regions, the explanatory power of climatic factors increased in cold and arid regions, also indicating that the effects of energy and water on species richness gradually increased with lower temperatures and the intensification of drought.

Min temperature of coldest month (BIO6) made the highest contribution to the species richness of *Quercus* in China. Sufficient energy, especially during the growing season and winter, is commonly regarded as a dominant factor limiting the growth and distribution of plants (Koehler et al., 2012; Larjavaara, 2014; Chen et al., 2021). Bhatta et al. (2021) confirmed that thermal energy was the core regulator for the species richness of plants in the Himalaya. Wang et al. (2011) explored the spatial distribution pattern of woody plants in China and its relationship with climatic factors. Their results clarified that mean temperature of coldest quarter (BIO11) had the greatest impact on species richness, and the distribution pattern of woody plants was mainly filtered by the frost. Most *Quercus* species originated from tropical or temperate zones, and the low temperature in winter would become one of the limitations for the northward migration of their distribution ranges (Sun et al., 2020). Among water variables, precipitation seasonality (BIO15) exhibited a high relative importance for species richness. Even though species of the *Quercus* genus are not highly dependent on water, unstable precipitation can still induce physiological damage to plants (Gao and Liu, 2018; Shrestha et al., 2018).

The tropics is widely considered the area with the highest biodiversity (Brown, 2014). However, environmental heterogeneity, historical events, and biological evolution have gave rise to diverse habitat preferences for different species (Chen et al., 2015; Sosa and Loera, 2017; Tripathi et al., 2019). The present research showed that the richness distribution hotspot of the *Quercus* genus in China was in the Hengduan Mountains, but the conditions of energy and water in this area were not the best. In terms of environmental heterogeneity, the uplift of the Qinghai–Tibet Plateau has led to complex topography and diverse vertical climate types in the Hengduan Mountains, resulting in a variety of habitats for plants and allowing species with different ecological niches to coexist in these areas (Stein et al., 2014; Pandey et al., 2020). In the present study, it was deduced that the high habitat heterogeneity in this region shaped the high species richness, which might also be the reason for the high unexplained residuals in variation partition analysis. In subsequent research, all related factors should be considered to examine the main environmental factors forming the richness distribution pattern of the *Quercus* genus in China.

## 5 Conclusions

Using the GWR model and a variety of statistical analyses, the effects of energy and water on the species richness of the *Quercus* genus in China and the explanatory powers of different climatic factors were quantified. The results indicated that the spatial variations in energy and water were important for explaining the richness distribution pattern. As cold and drought intensified, the effects of energy and water on species richness gradually increased. The climate effects were relatively stronger in arid regions, suggesting that arid regions would be more vulnerable to future climate change. In addition, this research found that energy played a more important role in forming the richness distribution pattern of the *Quercus* genus in China. However, some limitations existed in this research, such as possible bias in the acquisition of species richness data and the relatively low resolution of climatic data. The inclusion of phylogenetic analysis and an increased number of biotic and abiotic factors are necessary for further research. Nevertheless, exploring how species richness responds to climate has important theoretical value and practical implications for understanding the formation mechanisms of biodiversity and predicting the impact of future climate change on biodiversity.

## Data availability statement

The original contributions presented in the study are included in the article/**Supplementary Material**. Further inquiries can be directed to the corresponding authors.

## Author contributions

SS: Conceptualization, Data curation, Formal Analysis, Funding acquisition, Investigation, Methodology, Writing – original draft. YaZ: Data curation, Formal Analysis, Methodology, Writing – review & editing. NW: Methodology, Writing – review & editing. WY: Formal Analysis, Methodology, Writing – review & editing. YiZ: Formal Analysis, Methodology, Writing – review & editing. HW: Supervision, Writing – review & editing. PF: Investigation, Writing – review & editing. CY: Investigation, Writing – review & editing. PZ: Funding acquisition, Supervision, Writing – review & editing. RW: Funding acquisition, Supervision, Writing – review & editing.
