# Supplementary material for: Changing effects of energy and water on the richness distribution pattern of the Quercus genus in China
Source: Front Plant Sci. 2024 Jan 17;15:1301395. doi: 10.3389/fpls.2024.1301395 (PMC10827969; doi:10.3389/fpls.2024.1301395)
Supplement: Supplementary file 1 [file DataSheet_1.pdf]

1. Checklist of study species

Table S1. Checklist of study species

Number	Name
1	*Quercus acrodonta*
2	*Quercus acutissima*
3	*Quercus aliena*
4	*Quercus aquifolioides*
5	*Quercus baronii*
6	*Quercus bawanglingensis*
7	*Quercus chenii*
8	*Quercus cocciferoides*
9	*Quercus dentata*
10	*Quercus dolicholepis*
11	*Quercus engleriana*
12	*Quercus fabri*
13	*Quercus franchetii*
14	*Quercus griffithii*
15	*Quercus guyavifolia*
16	*Quercus kingiana*
17	*Quercus lanata*
18	*Quercus lodicosa*
19	*Quercus malipoensis*
20	*Quercus mongolica*
21	*Quercus monimotricha*
22	*Quercus oxyphylla*
23	*Quercus palustris*
24	*Quercus phillyreoides*
25	*Quercus rehderiana*
26	*Quercus robur*

27	*Quercus semecarpifolia*
28	*Quercus senescens*
29	*Quercus serrata*
30	*Quercus setulosa*
31	*Quercus spinosa*
32	*Quercus tarokoensis*
33	*Quercus utilis*
34	*Quercus variabilis*
35	*Quercus yunnanensis*

2. Results of Spearman correlation analysis

A

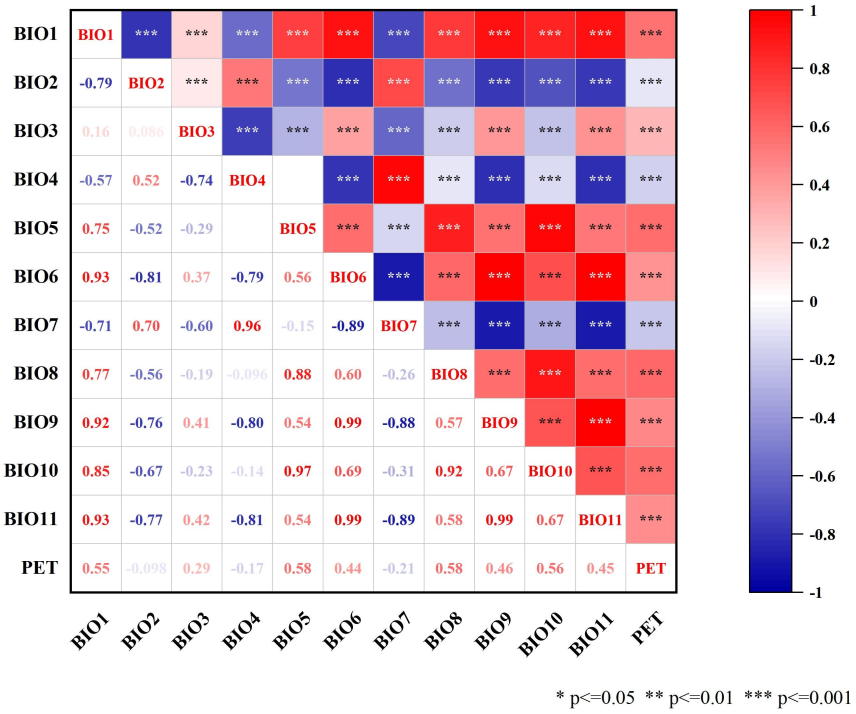

B

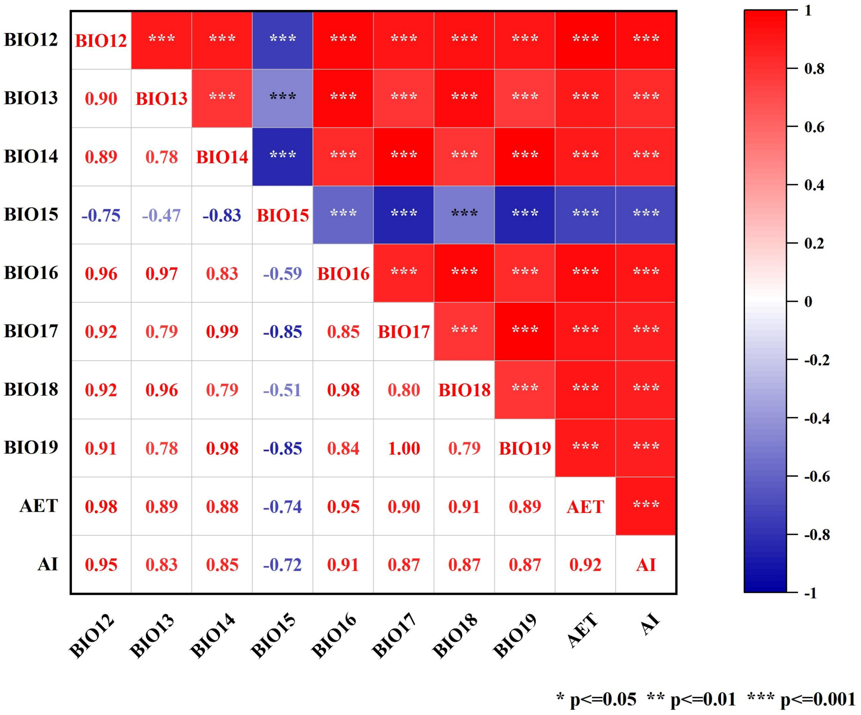

Figure S1. Heat map of Spearman correlation analysis. (A) Spearman correlation analysis of energy variables; (B) Spearman correlation analysis of

water variables. Red represents the positive correlation, while blue represents the negative correlation. Numbers represent the correlation coefficients, and * represents the significant correlation.**

3. Results of variation partition analysis

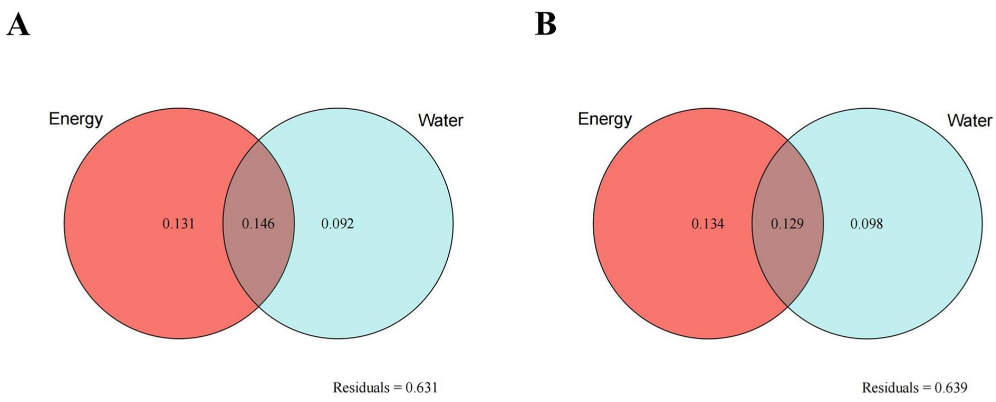

Figure S2. Results of variation partition analysis for the species richness of the *Quercus* genus in China with two sets of variables: energy (red circles) and water (blue circles). (A) Hot regions; (B) humid regions. Numbers represent the proportion of variation explained (%) by energy and water. The number in the intersection of the two circles represents the joint effect of energy and water. Residuals represent the unexplained variation.
